# Associations of changes in physical activity and sedentary time with weight recurrence after bariatric surgery: a 5-year prospective study

**DOI:** 10.1038/s41366-023-01284-7

**Published:** 2023-02-24

**Authors:** C. Sundgot-Borgen, D. S. Bond, F. F. Sniehotta, I. L. Kvalem, B. H. Hansen, I. Bergh, Ø. Rø, T. Mala

**Affiliations:** 1 Regional Department for Eating Disorders, Division of Mental Health and Addiction, Oslo University Hospital, Oslo, Norway; 2 Department of Surgery and Research, Hartford Hospital, Hartford, CT USA; 3 Population Health Science Institute, Faculty of Medical Sciences, Newcastle University, Newcastle upon Tyne, UK; 4 Department of Public Health, Preventive and Social Medicine, Medical Faculty Mannheim, Heidelberg University, Mannheim, Germany; 5 Department of Psychology, University of Oslo, Oslo, Norway; 6 Department of Public Health, Sport and Nutrition, University of Agder, Kristiansand, Norway; 7 Nudgelab, Oslo, Norway; 8 Institute of Clinical Medicine, University of Oslo, Oslo, Norway; 9 Center for Morbid Obesity and Bariatric Surgery, Oslo University Hospital, Oslo, Norway

**Keywords:** Weight management, Obesity

## Abstract

**Background:**

Increasing physical activity and limiting sedentary time may minimize weight recurrence after bariatric surgery. However, few studies have evaluated potential associations of objectively-measured physical activity and sedentary time with post-surgical weight recurrence over time.

**Aims:**

To evaluate associations of change in physical activity and sedentary time with weight recurrence after bariatric surgery.

**Methods:**

Participants from the Oslo Bariatric Surgery Study, a prospective cohort study, wore an ActiGraph monitor for seven days at 1- and 5 years after surgery to assess daily physical activity and sedentary time. Participants’ weight was measured at in-person clinic visits. Chi-square Test and Paired-samples T-test evaluated group differences and change over time, while Pearson’s Correlation, multiple logistic and linear regression investigated associations between variables.

**Results:**

Five years after surgery 79 participants (70.5% response rate, 81% female) (mean (sd) age: 54.0 (±9.3), BMI: 32.1 (±4.7)) had valid monitor data. Participants increased their sedentary time (71.4 minutes/day (95% CI: 54.2–88.6, *p* = <0.001)) and reduced daily steps (−1411.1 (95% CI: 737.8–208.4), *p* = <0.001), light physical activity (−54.1 min/day (95% CI: 40.9–67.2, *p* = <0.001)), and total physical activity (−48.2 (95% CI: 34.6–63.3), *p* = <0.001) from 1- to 5 years after surgery. No change was found for moderate-to-vigorous intensity physical activity. No associations were found between changes in steps, physical activity or sedentary time and weight recurrence.

**Conclusion:**

Participants increased sedentary time and decreased light- and total physical activity between 1- and 5 years post-surgery. Overall, changes in physical activity and sedentary time were not associated with weight recurrence. Interventions to help patients increase physical activity and limit sedentary time after bariatric surgery are needed.

## Introduction

Obesity is a highly prevalent, debilitating, and costly chronic disease [1, 2]. Bariatric surgery is the most effective treatment, and usually results in significant weight reduction, ranging from 25%–35% of initial weight [3, 4], along with improvements of obesity-related diseases [4, 5]. However, 30% of the patients experience weight recurrence up to 23.8% of maximum weight lost, 3–6 years after surgery [6–9]. This can lead to reemergence of obesity-related comorbidities and health-related quality of life impairments, as well as overall reduced satisfaction with the surgery performed [6–8]. Thus, it is important to identify modifiable factors that may prevent or reduce weight recurrence.

Among several factors that predict weight (e.g. metabolism, psychological and somatic issues, and eating habits), physical activity (PA) is a key strategy to enhance weight loss and prevent weight recurrence [10–16]. First, PA contributes to daily energy expenditure [17, 18]. PA can also help prevent decreases in resting energy expenditure via preservation of lean body mass which usually accounts for ~30% of weight loss following surgery [19]. Further, a recent study found that PA may assist with eating and appetite regulation in bariatric surgery patients [20].

Independent of PA, sedentary time (ST) has been identified as a predictor of mortality related to chronic disease [21]. Efforts to reduce ST among bariatric surgery patients may thus also be important [13]. ST might influence weight recurrence independent of or in conjunction with PA, however these associations have received less attention.

Due to absence of PA guidelines for bariatric surgery patients specifically, patients are often advised to follow national or international guidelines that recommend at least 150 min of moderate-vigorous physical activity (MVPA) (or 75 min of vigorous intensity) per week. Also, sitting more than 6–8 hours per day (h/day) has been related to increased metabolic risk [21], and guidelines recommend both sitting less and replacing time spent sedentary with movement [22].

While patients report changes in PA and ST after surgery, studies show that most patients do not meet recommendations for PA and ST one year after surgery according to objective measures [13, 14, 23, 24]. With subjective measures being prone to recall and other forms of bias, these findings highlight the importance of using objective measures to capture the true PA level and ST in individuals [25–29].

Whether objectively-measured PA and ST change over time beyond the typical weight loss period of 1–2 years [30] is less clear. Bellicha et al. found no changes in neither MVPA nor ST from pre-surgery to 5 years follow-up [31]. Launius et al. [24] found that daily steps and minutes in MVPA increased from surgery to 5 years follow-up [24]. This was supported by the findings of King et al. that showed that mean steps per day was higher at 5- and 7-years compared to pre-surgery. King et al. additionally reported that the percentage of participants meeting PA recommendations and total minutes in MVPA increased the two years following surgery. Despite a decline, these percentages were still higher compared to pre-surgery at 5 years follow-up, but lower compared to pre-surgery at 7-years. No reduction in ST was observed from pre-surgery through 5- and 7-years after surgery [14].

At 5 years, nearly all patients have experienced some amount of weight recurrence [6, 7, 9], thus it is important to look at whether variability in current PA and ST levels relate to the amount of weight recurrence. Few studies have examined this relationship, and even fewer have focused on ST. Bellicha et al. measured PA before, 6 months and 5 years after surgery and found no association between total PA and weight recurrence, but found a negative correlation between MVPA and weight recurrence, 5 years after surgery [31]. Nymo et al. assessed PA and weight recurrence 10–15 years after surgery and found no associations with steps per day, but a negative association between PA duration and weight recurrence [32]. Romagna et al. found that patients who regained less than 20% of initial weight loss had more steps and minutes in MVPA per day and were less sedentary compared to those who experienced ≥20% weight recurrence when both weight and PA was assessed simultaneously ≥5 years after surgery [13]. These findings were supported by King et al. who found that total steps per day and ST, but not MVPA, were associated with weight recurrence when assessed seven years after surgery [14]. If less sitting is associated with less weight recurrence to the same extent as being active, it might be important to focus on motivating patients to reduce ST especially among those who lack motivation or ability to meet PA recommendations.

Since patients on average are shown to make modest improvements in PA 1-year after surgery, and are still losing weight [13, 14, 23], it is important to understand how the magnitude of change in PA from 1-year to 5 years relates to weight recurrence. Studies find that changes in self-reported PA over time may associate more strongly with weight change following surgery, compared to cross-sectional PA [33, 34]. Thus, it is important to understand whether current objectively-measured PA levels and/or the change in PA levels over time matter for weight recurrence.

Weight recurrence may vary according to how MVPA is accumulated. Previous national guidelines recommended that MVPA should be accumulated in bouts of at least 10 min in duration [35]. However, the revised guidelines do not place any restrictions on how MVPA is accumulated [36]. Whether the pattern of MVPA accumulation is important for preventing weight recurrence has previously not been investigated. Also, only evaluating bouted minutes in MVPA might exclude many individuals within the bariatric surgery population because many do not engage in prolonged bouts of MVPA [27]. Importantly, studies within the general population find that using total minutes or bouted minutes in MVPA influence how participants are categorized [37–39]. Similar data is not available within the post-bariatric surgery population.

Thus, we aimed to evaluate whether both PA and ST at 5 years and changes in PA and ST from 1- to 5 years post-surgery are associated with weight recurrence 5 years after surgery. Additionally, we sought to investigate changes in objectively-measured PA and ST from 1- to 5 years after bariatric surgery and describe whether frequency of meeting PA recommendations differed based on the use of bouted minutes (≥10 min) or total minutes in MVPA.

## Method

### Participants and study design

The Oslo Bariatric Surgery Study (OBSS) [40, 41] is a prospective cohort study of patients admitted to bariatric surgery, either Sleeve gastrectomy or Roux-en-Y gastric bypass, at the Center for Morbid Obesity and Bariatric Surgery at Oslo University Hospital, Norway, from 2011 to 2013. The institution is a tertiary referral center for bariatric surgery operating some 300 patients annually. The 1991 National Institutes of Health Consensus Development Conference Statement for indication of bariatric surgery were applied throughout the study period. Clinical follow-up consultations were part of the post-surgery program and scheduled for all patients at 6 months, 1-, 2-, 3-, and 5 years after surgery. As part of the OBSS, objectively-measured PA and ST was investigated at 1- and 5 years after surgery. Recruitment procedure among the OBSS participants for the PA sub-study 1-year after surgery has been described previously [42].

All participants who accepted to wear an ActiGraph 1-year after surgery and returned valid data (*N* = 112) were eligible to take part in this current 5-year follow-up study. Respondents were invited to medical follow-up (mean (SD) years after surgery was 5.13 (0.34)) and additionally contacted and asked to wear an ActiGraph GT3X+ accelerometer for seven consecutive days after the medical follow-up (mean (SD) years between surgery and use of ActiGraph was 7.88 (0.75)). Data were collected in 2020, during the COVID-19 pandemic.

### Measures

Patients reported their age, employment status, educational level, and whether they had a partner or were married. Weight was measured using a calibrated Seca 635 III (0–300 kg) platform scale at follow-up consultations at 1-, 2-, 3-, and 5 years after surgery, with participants wearing light clothing and no shoes. For three participants, self-reported weight pre-surgery was used because objective measured weight was not obtained. Self-reported and objective weight pre-surgery was highly correlated (*r* = 0.96, *p* = <0.001), and self-reported values were therefore considered valid for these three individuals. Weight loss from day of surgery to 5 years follow-up was calculated as %TWL = [(Weight on the day of surgery) − (Postoperative Weight)]/(Weight on the day of surgery) × 100 [43], and based on suggested successful weight loss, weight loss was further categorized as ≥20% and <20% of pre-surgery weight [44]. Nadir weight was defined as the lowest postoperative body weight objectively assessed at 1-, 2-, 3- and 5 years after surgery. Weight recurrence was used as a continuous and dichotomous variable, and calculated by 100*(Weight at 5 years follow-up—nadir)/(pre-surgery weight—nadir). Weight recurrence was chosen to be investigated in relation to PA and ST, as it is the weight variable most strongly correlated with cardiometabolic outcomes in bariatric surgery patients [45]. For the same reason as for %TWL, weight recurrence has been recommended to be dichotomized into ≥20% or <20% of maximal weight loss [45]. Body mass index (kg/m^2^) (BMI) was calculated at the day of surgery and at medical follow-up consultations at 1- and 5 years after surgery.

### Objectively-measured physical activity

The ActiGraph GT3X+ activity monitor (ActiGraph, LLC, Pensacola, FL, USA) was used to measure PA and ST. The participants were instructed to wear the accelerometers on their right hip during all waking hours for seven consecutive days, except during showering and bathing. ActiGraph data was included into analyses if containing at least four days with more than 10 h of valid data per day. The accelerometer data was used to assess steps per day, mean counts per minute (cpm), sedentary time (0–99 cpm), light- (100–2019 cmp), moderate- (2020–5998 cpm) vigorous intensity (≥5999 cpm), and total PA (100->5999 cpm) [46, 47], and finally the percentage of the study population that met the current national PA recommendations of at least 150 min of MVPA (or 75 min of vigorous intensity) per week. Adherence to PA recommendations was determined by summing the time spent performing MVPA in two ways. First, we followed the previous guidelines on how to accumulate MVPA minutes which recommended to sum all minutes spent in continuous MVPA in bouts lasting at least 10 min (with allowance for two interruptions) (bouted MVPA) [35]. Second, we followed the 2018 Committee that suggested to sum all minutes spent in MVPA (total minutes in MVPA) [36]. Both guidelines on how to accumulate minutes, recommend a weekly MVPA of at least 150 min, which reflects the international guidelines for healthy adults published by WHO [22]. Regarding ST, both WHO and the Norwegian directory of health recommend to sit less [22, 48]. Furthermore, sitting more than 6–8 h/day has been related to increased metabolic risk [20], and participants were classified according to whether they accumulated 8 h or more of ST per day.

### Statistics

#### Attrition analysis

Logistic regression was used to examine explanatory factors for attrition to the 5-year follow-up study. Among independent variables (age, sex, employment status, education level, relationship status, BMI, weight- and weight loss, and objectively-measured PA and ST, at 1-year after surgery), only BMI and weight correlated with attrition, but showed high intercorrelation (*r* = >0.8). Hence, only weight 1-year after surgery was included into the regression model.

#### Statistical analyses

All data were analyzed with IBM SPSS version 26. Descriptive statistics are presented as mean (sd) and sum (%) for continuous and categorical data, respectively. After visually evaluating the data for normality we used a Chi-Square Test to investigate differences in frequency of participants meeting recommendations on PA and ST between 1- and 5 years after surgery. Paired-samples t-test was used to investigate changes in continuous PA and ST data from 1- to 5 years follow-up. Multiple regression analyses were used to control for possible confounders (sex, age, BMI, or time between 5-year medical follow-up and 5-year assessment of PA) on the change in steps, each PA intensity and ST, and investigate predictors for attrition from 1-year to 5 years participation. Prior to the linear regression, Pearson’s correlation was used to investigate correlation between the dependent and independent variables and for multicollinearity between the independent variables. A multiple logistic regression was chosen to investigate group differences in continuous PA and ST data between weight recurrence groups while controlling for possible confounders (age, sex, %TWL at 1-year after surgery, relationship status, employment status, and educational level). The predictive value of each independent variable is presented through Odds Ratio (OR) with 95% confidence intervals (CI). CIs that do not cross the value of 1 represent significance. For other analyses, *p*-values at ≤0.05 were evaluated as statistically significant. Hypothesis testing analyses were two-sided.

## Results

**Fig. 1:**
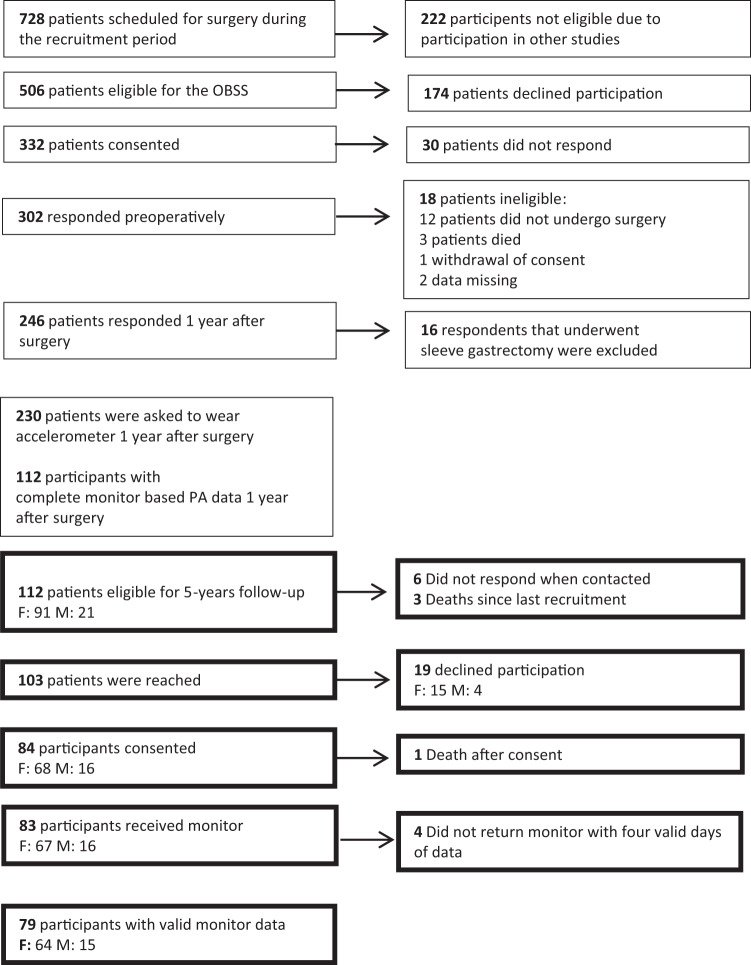
Flow chart of the recruitment process. The flow chart presents the number of individuals who were eligible for the study at 1- and 5-years after surgery, number of individuals reached, number of acceptance and decline, number of excluded individuals, resons for exclusion (boxes to the right-hand side), and final number of included participants in this study.

From the 112 participants who returned valid monitor data at 1-year follow-up, and therefore were eligible to be recruited to the 5-year follow-up, 79 (70.5%) participants consented to use the monitor and had four valid days of data at 5 years after surgery (Fig. 1). Among these, 30% at 1-year and 32.9% at 5-year returned monitors without any bouted minutes in MVPA. Attrition analysis found that a higher body weight at 1-year reduced the likelihood of participating at 5 years after surgery (odds ratio: 0.96, 95% CI: 0.93, 0.98).

### Demographics

Participant characteristics 5 years after surgery are shown in Table 1. After a reduction in mean (sd) body weight of 36.35 (11.07) kg from the day of surgery to 1-year after surgery, the participants gained a mean (sd) of 6.6 (9.13) kg (*p* = <0.001) from 1-year to 5 years after surgery. Time-point for nadir weight, reported as mode was at 1-year follow-up (min = 1, max = 5). The majority had lost ≥20% of preoperative weight, and half of the sample had ≥20% weight recurrence 5 years after surgery (Table 1).

**Table 1:** Participant characteristics 1- and 5 years after bariatric surgery.

Variables	Number (%) or Mean (sd)^a^			
	1-year after surgery (*n* = 79)		5 years after surgery (*n* = 79)	
Sex				
Male	–		15	(18.9%)
Female	–		64	(81.1%)
Roux-en-Y gastric bypass	–		78	(98.7%)
Sleeve gastrectomy	–		1	(1.3%)
Higher level of education^c^	–		26	(32.9%)
Age	46.4	(9.3)^a^	54.0	(9.3)^a^
Having a partner/married	56	(70.9%)	57	(79.2%)
Employed 100%	57	(72.2%)	37	(51.4%)
%TWL	29.7%	(9.5–42.9%)	24.1%	(3.4–43.2%)
<20%	7	(9.5%)	27	(35.1%)
≥20%	67	(90.5%)	50	(64.9%)
Weight recurrence^b^	–		24.3%	(−6.7–84.9%)
<20%	–		38	(49.4%)
≥20%	–		39	(50.6%)
BMI, kg/m^2^	29.9	(4.6)^a^	32.1	(4.7)^a^
Normal/underweight	8	(10.8%)	2	(2.6%)
Overweight	37	(50.0%)	22	(28.6%)
Obese1	17	(23.0%)	33	(42.9%)
Obese2	9	(12.2%)	14	(18.2%)
Obese3	3	(4.1%)	6	(7.8%)

The table presents 1-year follow-up data only for participants who were included at 5 years follow-up. Weight recurrence: weight recurrence as percent of maximum weight loss.

*%TWL* percent total weight loss.

^a^The number within the parenthesis is sd, and not %.

^b^Weight recurrence less or more than 20% of maximum weight loss. BMI categories: under/normal weight: <25, overweight: 25–29.9, obese grade 1: 30–34.9, Obese grade 2: 35–39.9, obesity grade 3: >40.

^c^University, college or the equivalent (education exceeding 12 years).

### Physical activity and sedentary time 5 years after surgery

**Fig. 2:**
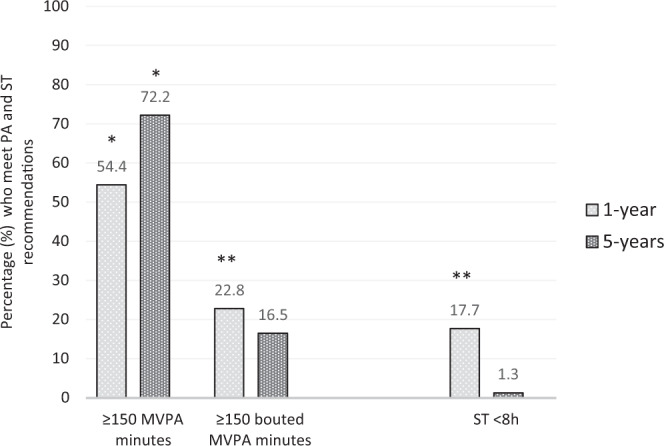
Percentage of 79 participants who meet physical activity and sedentary time recommendations 1- and 5 years after bariatric surgery. Note. Moderate-to-vigorous physical activity: MVPA. MVPA minutes: frequency of participants who meet physical activity (PA) recommendations based on total minutes per day in MVPA; Bouted MVPA minutes: frequency of participants who meet the PA guidelines based on total minutes spent in MVPA bouted in at least 10 min; ST sedentary time. The recommended level of PA according to the Norwegian national guidelines is 150 min objectively-measured [46]. *Significantly different percentage from the percentage meeting PA recommendations based on bouted MVPA. **Significantly different percentage from 1- to 5 years. α = ≤0.05. *n* = 79.

At 5 years participants spent on average 79% of waking hours being sedentary, and about 11 and 30 min in bouted MVPA and total minutes in MVPA, respectively (Table 2). Recommendations on PA level and ST were met by 16.5% and 1.3%, respectively (Fig. 2).

**Table 2:** Physical activity and sedentary time in 79 participants 1- and 5 years after bariatric surgery.

	1-year Mean (sd)		5-year Mean (sd)							
Physical activity level					Mean diff (sd)		CI 95%	*t*, (df = 78)	*p*	*d*
Counts per minute	292.4	(112.6)	261.6	(112.0)	30.8	(111.8)	5.8, 55.9	2.5	**0.017**	0.27
Steps per day	7402.9	(2737.6)	5991.8	(2828.8)	−1411.1	(3006.0)	737.8, 2084.4	4.2	**<0.001**	0.51
Sedentary time (min/day)	576.7	(84.0)	648.1	(74.6)	71.4	(76.9)	88.6, 54.2	8.3	**<0.001**	1.35
Light intensity (min/day)	220.2	(53.2)	166.1	(55.0)	−54.1	(58.7)	40.9, 67.2	8.2	**<0.001**	0.99
Moderate intensity (min/day)	27.7	(20.4)	33.4	(19.7)	−5.6	(22.9)	−10.8, −0.5	−2.2	**0.032**	0.28
Vigorous intensity (min/day)	1.3	(3.1)	0.8	(2.0)	−0.5	(2.4)	−0.1, 1.0	1.8	0.081	
Total PA (min/day)	249.2	(55.7)	200.3	(64.0)	−48.2	(64.2)	34.6, 63.3	6.8	**<0.001**	1.23
MVPA (min/day)	29.0	(21.9)	34.2	(20.7)	−5.2	(23.4)	−10.4, 0.1	−1.9	0.054	
Bouted MVPA (min/day)^a^	11.9	(15.0)	11.2	(14.5)	−0.7	(18.6)	−3.5, 4.9	0.3	0.740	

*Mean diff* Mean difference*, CI 95%* 95% confidence interval, *d* Cohen’s *d*, *PA* physical activity, *MVPA* moderate-to-vigorous physical activity, *Total PA* total physical activity (Light PA + MVPA).

A *p* value of ≤0.05 is set as statistically significant.

Bold values define statistically significant *p*-values.

^a^All activity <2020 cpm that occurred in sustained bouts of at least 10 min (with allowance for two drops in intensity).

### Changes in physical activity and sedentary time from 1- to 5 years after surgery

Participants increased their time spent sedentary and reduced daily steps, and time in light- and total PA from 1-year to 5 years after surgery (*p* = <0.001). No changes were found for total minutes in MVPA or bouted MVPA (Table 2). Multiple regression models did not indicate that changes over time were related to confounders such as gender, age, BMI, or time between medical follow-up and use of PA monitor. We found no change in percentage of participants who met PA recommendations based on total minutes in MVPA, while a lower percentage of participants met PA recommendations based on bouted minutes in MVPA at 5 years (16.5%) compared to 1-year (22.8%) after surgery (*φ* = 0.25, *p* = 0.028) (Fig. 2). A lower percentage of participants met the threshold recommendations for ST (<8 h per day) (*φ* = 0.24, *p* = 0.030) at 5 years (1.3%) compared to 1-year (17.7%) after surgery.

### Association between physical activity, sedentary time, and weight recurrence

No significant correlation was found between daily steps (*r* = −0.12, *p* = 0.289), different PA intensities (*r* = −0.21–0.07, *p* = 0.063–0.698) and ST (*r* = 0.05, *p* = 0.640) and weight recurrence at 5 years after surgery (Table 3). Nor did we find any associations between changes in steps (*r* = −0.12, *p* = 0.289), PA intensities (*r* = −0.05–0.11, *p* = 0.395–0.876) and ST (*r* = −0.04, *p* = 0.728) and weight recurrence (Table 4).When controlling for possible confounders, neither daily number of steps, minutes spent in different PA intensities or being sedentary, significantly predicted whether participants experienced ≥20% or <20% weight recurrence at 5 years after surgery (see Supplementary Table 1).

**Table 3:** Correlations between daily steps, physical activity intensities, sedentary time, and weight recurrence in 79 individuals 5 years after bariatric surgery.

	Weight recurrence at 5 years after surgery			
	*r*	95% CI		*p*
Steps per day	−0.12	−0.34	0.10	0.289
Sedentary time (min/day)	0.05	−0.17	0.28	0.640
Light PA (min/day)	−0.05	−0.27	0.18	0.698
Moderate PA (min/day)	0.07	−0.16	0.29	0.540
Vigorous PA (min/day)	−0.21	−0.42	0.01	0.063
Total PA (min/day)	−0.02	−0.25	0.20	0.839
Total min/day in MVPA	0.05	−0.18	0.27	0.687
Total min/day in bouted MVPA	0.01	−0.22	0.23	0.932

*PA* physical activity, *MVPA* moderate-to-vigorous physical activity, *Total PA* total physical activity (Light PA + MVPA), *95% CI* 95% confidence interval.

A *p* value of ≤0.05 is set as statistically significant.

**Table 4:** Correlations between changes in daily steps, physical activity intensities, sedentary time, and weight recurrence in 79 individuals from 1–5 years after bariatric surgery.

	Weight recurrence at 5 years after surgery			
	*r*	95% CI		*p*
Change in steps per day	−0.12	−0.18	0.27	0.289
Change in sedentary time (min/day)	−0.04	−0.26	0.19	0.728
Change in light PA (min/day)	0.02	−0.21	0.24	0.876
Change in moderate PA (min/day)	0.10	−0.13	0.31	0.413
Change in vigorous PA (min/day)	0.06	−0.17	0.28	0.625
Change in total PA (min/day)	−0.05	−0.27	0.17	0.649
Change in total min/day in MVPA	0.20	−0.13	0.32	0.395
Change in total min/day in bouted MVPA	0.11	−0.12	0.32	0.360

*PA* physical activity, *MVPA* moderate-to-vigorous physical activity, *Total PA* total physical activity (Light PA + MVPA), *95% CI* 95% confidence interval.

A *p* value of ≤0.05 is set as statistically significant.

### Total minutes in MVPA and minutes in bouted MVPA

When comparing methods of accumulating minutes in MVPA, we found that a higher percentage of participants met PA recommendations when referring to total minutes in MVPA compared to bouted minutes in MVPA (*φ* = 0.49, 0.28 *p* = <0.001, 0.014, 1- and 5 years, respectively) (Fig. 2).

## Discussion

This is one of the first study to evaluate associations of long-term changes in PA and ST with weight recurrence after bariatric surgery in a Scandinavian patient sample. We found that participants on average did not make favorable changes in PA or ST. Neither PA and ST at 5 years nor changes in PA and ST over time were associated with weight recurrence overall.

### Changes in physical activity level and sedentary time

In accordance with previous studies in Brazil, USA, and Sweden [13, 14, 23, 24] we found that participants did not meet PA recommendations and exceeded the suggested threshold (<8 h) for ST one year after surgery. Furthermore, we found that participants on average became even more sedentary, had fewer steps, spent less time in light- and total PA and fewer met MVPA recommendations 5 years after surgery. This reflects a negative trend over time. Our MVPA findings support Bellicha et al. who found no change in MVPA 5 years after surgery [31], contradict the significant increase in steps and minutes in MVPA reported by Launius et al. [24], and partly King et al. who reported a small increase in steps over time, whereas MVPA increased at 5 years which subsequently decreased at 7-years [14].

Our participants increased ST over time in contrast to no change reported by Bellicha et al. and King et al. Intuitively, patients might be expected to increase their PA level and reduce their ST after surgery due to substantial weight loss and improved physical function. However, our findings based on 5-year follow-up data, which align with those from previous studies, do not support this theory. The gap between current recommendations and participants’ PA level and ST reflects an increased risk for chronic disease mortality [21], and poorer overall long-term health [15, 16]. In our sample, the mean difference and the effect size was significantly larger for the increase in ST and reduction in light PA, compared to changes in other PA intensities. Therefore, from a health behavior perspective, it might be most beneficial to intervene on these behaviors if the aim is to comply with PA and ST recommendations. Evidence also suggests that although there is a dose-response relationship between all-cause mortality and PA level, the strongest effect is reported when inactive individuals move towards sitting less and engaging in light intensity activity [49].

### Association with weight recurrence

No significant association was found between daily steps or any PA intensity at 5 years or changes in steps and PA intensities over time, with weight recurrence at 5 years after surgery, in our sample. This was also reflected by the lack of difference between weight recurrence groups in steps and PA intensities. This contradicts the negative association found between steps and weight recurrence, but is in line with findings of no association with MVPA, reported by King et al. Our findings related to steps also echoes findings by Nymo et al. but contradicts the negative correlation between PA and weight recurrence ≥5 years after surgery reported by both Bellichia et al. and Nymo et al. [31, 32].

We found no significant association between ST at 5 years or changes in ST over time, with weight recurrence at 5 years after surgery. No difference was neither found between those who experienced ≥20% and <20% weight recurrence. The lack of associations at 5 years contradicts the positive association reported by King et al. in participants 7 years after surgery, however the lack of association when looking at changes in ST over time were similar between the two studies [14]. Because most of our participants exceeded the recommended ST threshold (>8 h), we were statistically not able to investigate group differences related to meeting or not meeting ST recommendations and associations with weight recurrence.

When examining the changes in PA and ST over time, the changes in minutes spent in PA intensities and ST reported in our sample could be too small to have an impact on weight recurrence, and the magnitude of associations might appear different in samples where change over time are more prominent [10–13, 15, 16]. When interpreting our correlational data, it is important to have in mind that the correlations for both 5 years and changes from 1–5 years are characterized by large 95%CIs, with *e.g*. r-values ranging from *r* = −0.2–0.2. This indicates that the sample size might negatively impact the ability to present a precise correlation between variables.

### Total minutes in MVPA vs. bouted MVPA

At 5 years after surgery, 72.2% of participants met current MVPA recommendations (≥150 MVPA minutes/week) based on total minutes in MVPA but only 16.5% of participants met previous MVPA recommendations based on bouted MVPA minutes. These data are aligned with findings within the general adult population [37–39], but represent novel information on the bariatric surgery population. Choosing the one method over the other clearly decides if our participants are described as a high-risk group for chronic diseases or not, and whether their PA level compares to, or exceeds, the PA level of the general adult population.

Using all minutes in MVPA might be beneficial when evaluating participants following bariatric surgery from several perspectives. Shorter bouts (<10 min) may be more realistic and easily incorporated into the daily life post-surgery, and more clearly reflect movement through an active lifestyle [29]. This activity is not captured with bouted minutes. Also, ~30% of our sample did not participate in any bouted MVPA. Hence, only considering bouted MVPA minutes excludes a large group of participants from being evaluated on an important health-related variable. Namely, using all minutes in MVPA has been found to similarly associate with the metabolic syndrome [39, 50] and cardiometabolic biomarkers when compared to bouted MVPA [51], and could provide equally important information in terms of whether a patient needs to improve MVPA to maintain health.

### Strengths and limitations

Among study strengths are: (1) the long-term assessments, (2) the use of objective measures, (3) the additional focus on ST, (4) the investigation of associations between both PA and ST with weight recurrence in a long-term perspective, and (5) the methodological exploration of all minutes in MVPA and bouted minutes in MVPA. Some analyses were most likely underpowered, which indicated that possible group differences and associations related to weight recurrence may have been underestimated. Reported nadir weight depended on weight assessed at time-specific clinical follow-ups, and could therefore differ slightly from the true nadir weight experienced in-between follow-ups. Due to only one sleeve gastrectomy patient, we could not evaluate if results differed by surgery procedure. Also, in accordance with most bariatric surgery studies, our results mainly reflect the female population. Finally, data were collected during the COVID-19 pandemic which could impact our findings since social isolation during the pandemic associated with less PA activity and more ST among previous bariatric surgery patients [52].

### Research and practical implications

For the average patient, improvement of PA and a reduction in ST is beneficial for promoting health and weight related benefits of the surgery. Focusing on replacing ST with light PA activity might be more acceptable and easier initially for some patients than to mainly focus on an increase in MVPA. An additional encouragement to increase MVPA to achieve larger increases in overall daily movement as per national guidelines is important. Future research should focus on both long-term PA level and ST within a larger sample size to improve our understanding of their combined and separate influence on weight recurrence. Interventions to help bariatric surgery patients increase PA and limit ST for overall health are needed. However, to better evaluate whether changes in PA and ST influence weight recurrence, randomized controlled trials, in which true changes in behaviors are observed and possible confounders are controlled for, are needed [53]. It might be appropriate to suggest an additional focus on all minutes in MVPA to include all participants and to evaluate PA more appropriately among this specific population.

## Conclusion

In this study the participants were overall less active and more sedentary at 5 years compared to 1-year after bariatric surgery. No statistically significant associations were observed between continuous measures of PA and ST and weight recurrence.

## Supplementary information

Supp Table 1

## Data Availability

The dataset analyzed during the current study is available from the corresponding author on reasonable request.
