# Supplementary material for: Associations of changes in physical activity and sedentary time with weight recurrence after bariatric surgery: a 5-year prospective study
Source: Int J Obes (Lond). 2023 Feb 24;47(6):463–70. doi: 10.1038/s41366-023-01284-7 (PMC9951836; doi:10.1038/s41366-023-01284-7)
Supplement: Supplementary file 1 — Supp Table 1 [file 41366_2023_1284_MOESM1_ESM.docx]

# Supplementary file

Table 1. Logistic regression presenting likelihood of experiencing ≥20% or <20% weight recurrence at 5-years follow-up

|  | **B** | **S.E.** | **Wald** | **df** | ***p*** | **OR** | **95% CI for OR** | |
| --- | --- | --- | --- | --- | --- | --- | --- | --- |
| Age | -0.01 | 0.04 | 0.06 | 1 | .811 | 0.99 | 0.92 | 1.07 |
| Sex | -0.35 | 0.81 | 0.18 | 1 | .668 | 0.71 | 0.14 | 3.47 |
| % weight loss 1-y post-surgery | -0.02 | 0.04 | 0.29 | 1 | .594 | 0.98 | 0.90 | 1.06 |
| Partner/married | -0.81 | 0.97 | 0.70 | 1 | .404 | 0.45 | 0.07 | 2.98 |
| 100% Employed | 0.35 | 0.76 | 0.22 | 1 | .643 | 1.42 | 0.32 | 6.27 |
| Higher level of education^b^ | 0.31 | 0.69 | 0.20 | 1 | .653 | 1.37 | 0.35 | 5.38 |
| Daily Steps | -0.00 | 0.00 | 4.40 | 1 | .036 | 0.99 | 0.99 | 1.00 |
| Sedentary time (min/day) | 0.00 | 0.01 | 0.15 | 1 | .701 | 1.00 | 0.99 | 1.01 |
| Light PA (min/day) | 0.02 | 0.01 | 1.92 | 1 | .166 | 1.02 | 0.99 | 1.04 |
| Moderate PA (min/day) | 0.08 | 0.04 | 5.11 | 1 | .024 | 1.08 | 1.01 | 1.16 |
| Vigorous PA (min/day) | -0.38 | 0.34 | 1.25 | 1 | .263 | 0.69 | 0.35 | 1.33 |
| Bouted MVPA (min/day) | 0.02 | 0.04 | 0.21 | 1 | .650 | 1.02 | 0.94 | 1.11 |
| Constant | -0.30 | 4.74 | 0.00 | 1 | .950 | 0.74 |  |  |

Note. ^b^University, college or the equivalent (education exceeding 12 years). PA= physical activity. MVPA= moderate to vigorous physical activity. Total PA: total physical activity (Light PA + MVPA). OR= odds ratio. A *p*-value of ≤0.05 is set as statistically significant, but 95% CI for OR which crosses the value 1 is not considered statistically significant.

The full model containing all predictors was not statistically significant. This was defined by the goodness of fit test, represented by the Omnibus Tests of Model Coefficients *X*^2^ (12, n= 61)= 15.67, *p*= .207. Although daily number of steps and minutes spent in moderate PA significantly predicted the likelihood of experiencing more or less than 20% weight recurrence five years after surgery based on *p*-values, the 95%CI of the OR crossed the value of 1. No significant difference in PA intensities and sedentary time was therefore observed between the ≥20% or <20% weight recurrence groups 5-years after surgery.
